# Amygdala Enlargement in Patients with Mesial Temporal Lobe Epilepsy without Hippocampal Sclerosis

**DOI:** 10.3389/fneur.2013.00166

**Published:** 2013-10-25

**Authors:** Ana Carolina Coan, Marcia Elisabete Morita, Brunno Machado de Campos, Clarissa Lin Yasuda, Fernando Cendes

**Affiliations:** ^1^Neuroimaging Laboratory, Department of Neurology, State University of Campinas, Campinas, Brazil

**Keywords:** amygdala, temporal lobe epilepsy, MRI-negative, volumetry, voxel-based morphometry

## Abstract

**Purpose:** Patients with mesial temporal lobe epilepsy (MTLE) without MRI abnormalities (MTLE-NL) represent a challenge for definition of underlying pathology and for presurgical evaluation. In a recent study we observed significant amygdala enlargement (AE) in 14% of MTLE patients with MRI signs of hippocampal sclerosis. Areas of gray matter volume (GMV) increase could represent structural abnormalities related to the epileptogenic zone or part of a developmental abnormality. Our objective was to look for undetected areas of increased GMV in MTLE-NL using post processing MRI techniques to better understand the pathophysiology of this condition.

**Methods:** We evaluated 66 patients with MTLE-NL on visual analysis and 82 controls. Voxel-based morphometry (VBM) group analysis was performed with VBM8/SPM8 looking for areas of increased GMV. We then performed automatic amygdala volumetry using FreeSurfer software and T2 relaxometry to confirm VBM findings.

**Results:** Voxel-based morphometry group-analysis demonstrated increased amygdala volume in the MTLE-NL group compared to controls. Individual volumetric analysis confirmed AE in eight (12%) patients. Overall, from all patients with AE and defined epileptic focus, four (57%) had the predominant increased volume ipsilateral to the epileptic focus. These results were cross-validated by a secondary VBM analysis including subgroups of patients according to the volumetric data. T2 relaxometry demonstrated no amygdala hyperintense signal in any individual with significant AE. There were no clinical differences between patients with and without AE.

**Discussion:** This exploratory study demonstrates the occurrence of AE in 12% of patients with MTLE-NL. This finding supports the hypothesis that there might be a subgroup of patients with MTLE-NL in which the enlarged amygdala could be related to the epileptogenic process. Further studies are necessary but this finding could be of great importance in the understanding of MTLE-NL.

## Introduction

Mesial temporal Lobe Epilepsy (MTLE) is frequently associated with hippocampal sclerosis (HS), however there is a significant group of patients with MTLE that do not have MRI signs of HS nor other lesions on MRI visual analysis, the so called “MRI-negative” patients (1).

Mesial temporal lobe epilepsy with normal MRI (MTLE-NL) is a very challenging condition, especially when patients are under evaluation for epilepsy surgery (2, 3). Patients with MTLE-NL often show a different course of the disorder and worse surgical outcome than patients with HS (4). MTLE-NL can be considered a different syndrome from MTLE with HS (5). For MTLE-NL patients it is still unknown whether there is lack of structural abnormality or if there is an underlying cause that we still do not understand.

Although no obvious epileptogenic lesion is detected on MRI, many of these patients undergo temporal lobectomy after appropriate presurgical evaluation (6–8) that usually includes prolonged EEG recordings with intracranial electrodes. These procedures usually carry some risks and complications (9). Thus, the use of non-invasive techniques looking for the possible underlying cause of seizures in these patients could help the improvement of surgical treatment. The use of post-process techniques to evaluate subtle structural abnormalities in MR imaging of patients with MTLE-NL have been used to look for areas of gray matter atrophy (2). However, areas of subtle diffuse atrophy can be related to secondary damage rater than to the epileptogenic zone. On the other hand, areas of gray matter increase could correlate better with the epileptic focus in these patients. Indeed, in recent years, it has become clear that MRI-negative epilepsies are not necessarily non-lesional, the primary histopathological substrate being subtle focal cortical dysplasia (FCD) (3, 10) which could be possibly identified as subtle gray matter increase in specific post-process MRI techniques (10, 11).

In a recent study of 102 MTLE patients with MRI signs of HS, we observed significant amygdala enlargement (AE) in 14 (14%) individuals (12). Areas of gray matter increase in these individuals could represent structural abnormalities related to the epileptogenic zone or part of a developmental abnormality.

This exploratory study was designed to look for areas of subtle gray matter increase in a group of MTLE-NL and to correlate these findings with the clinical characteristics in these patients, in order to better understand the pathophysiology of this condition. We opted to perform initially a group analysis with the hypothesis that we would be able to observe abnormalities of subgroups of patients. As a secondary analysis, individual volumetry was performed to isolate the patients in whom the abnormalities were present.

## Materials and Methods

### Patients’ selection

We included 66 patients with mean age of 41 years, [standard deviations (SD), ±12.2 years, ranging from 19 to 74 years, 39 female] who had clinical and electroencephalographic diagnosis of MTLE with normal MRI on visual analysis and hippocampal volumetric analysis. Thirteen patients (20%) were free of seizures for at least 2 years and 53 (80%) had antiepileptic drug (AED) resistant seizures. Patients were followed at the Epilepsy Clinic of the University of Campinas (UNICAMP). Prior to acquisition of MRI data all patients signed an informed consent form approved by the Ethics Committee of UNICAMP. For the neuroimaging analysis we acquired 3D images, sagittal T1-weighted, with voxel size of 1 mm × 1 mm × 1mm (TR = 7 ms, TE = 3.2 ms, flip angle = 8°, matrix = 240 × 240) in a 3 T MRI scanner (Philips Medical Systems, Best, The Netherlands) of all patients and of a control group of 82 healthy subjects. All images underwent visual inspection and only patients with normal MRI by visual analysis were selected. To increase the specificity of the visual MRI analysis and to exclude the individuals with subtle HS signs, we also performed automatic hippocampal volume measurements in all patients and in 82 healthy subjects using FreeSurfer software (version 5.1.0^1^). Hippocampal volumes were corrected for brain volumes of each individual. Patients with hippocampal volumes lower than 2 SD (absolute value and/or asymmetry index, defined by the ratio of smaller over the larger hippocampus of each individual) from the mean of the control group were excluded from this analysis. We collected clinical information regarding sex, age, side of epileptic focus, age of seizure onset, time of epilepsy, history of initial precipitate injury, or *status epilepticus*, family history of epilepsy, occurrence of generalized tonic-clonic seizures (GTCS), frequency of complex partial seizures (CPS), and GTCS in the previous year.

### Voxel-based morphometry

For voxel-based morphometry (VBM) we used the acquired 3D images for patients and for a control group of 82 healthy subjects. Pre-processing and statistical analysis were performed with VBM8/SPM8 toolbox. The resultant gray matter images were smoothed to remove large signals discrepancies between neighboring voxels (10 mm FWHM). A test of quality was performed to observe homogeneity and co-registration between the data and outliers were excluded from this study (three controls and four with MTLE-NL). A two-sample *t*-test (*p* < 0.001, minimum threshold cluster of 30 voxels) was performed between MTLE-NL and controls.

### Amygdala volumetry

To confirm VBM findings we performed amygdala volume (AV) measurements in all patients and in 82 healthy subjects using FreeSurfer software. AVs were corrected for brain volumes of each individual. Patients with AVs lower or higher than 2 SD (absolute value and/or asymmetry index, defined by the ratio of smaller over the larger amygdala of each individual) from the mean of the control group were considered abnormal. We also analyzed a subgroup of patients with AVs between 1.5 and 2 SD from the mean of controls.

We compared right and left AVs with the control group using a *t*-test.

### T2 relaxometry

T2 relaxometry of amygdala was performed in patients with significant increase or decrease of amygdala volume to investigate signal abnormalities (higher than 2 SD of the mean of the control group, composed by 82 healthy subjects). For this analysis we used T2 multi-eco images (3 mm slices; TR = 3300; TE = 30/60/90/120/150; matrix = 200 × 176; FOV = 1802 × 180) and the in-house software Aftervoxel^2^.

### Cross-validation VBM analysis

In order to cross validate the VBM and volumetry we performed a secondary VBM analysis based on groups defined by amygdala volumetry. Because the binary definition of abnormally enlarged (>2 SD from controls) and normal (within 2 SD from controls) AVs may include some individuals with less pronounced enlargement, we included a third subgroup as follows:

1. MTLE-NL with AE higher than 2 SD from the mean of the control group (enlarged amygdala volume).
2. MTLE-NL with AVs higher than 1.5 SD from the mean of controls (borderline AE).
3. MTLE-NL with AVs lower than 1.5 SD from the mean of controls (normal amygdala volume).

Pre-processing and statistical analysis were performed with VBM8/SPM8 toolbox. The resultant gray matter images were smoothed to remove large signals discrepancies between neighboring voxels. A two-sample *t*-test (*p* < 0.05, FDR corrected; minimum threshold cluster of 30 voxels) was performed between the described group and controls.

## Results

### VBM group analysis

**Figure 1:**
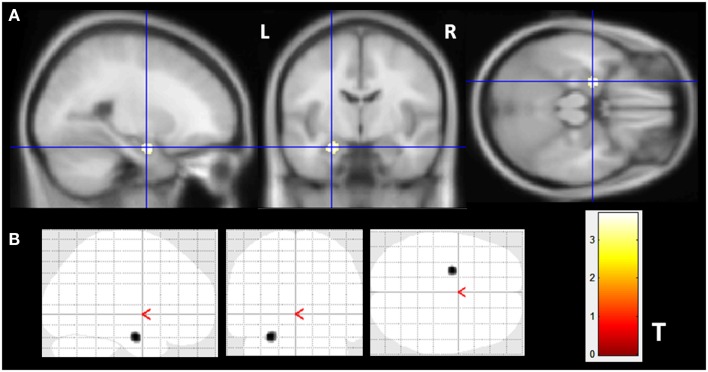
**Gray matter volume increase in patients with MTLE-NL**. VBM analysis looking for matter volume increase in patients with MTLE-NL demonstrated significant amygdala enlargement on the left side **(A,B)** – glass brain view. VBM, Two-sample *t*-test, *p* < 0.001, minimum cluster size of 30 voxels. MTLE-NL, mesial temporal lobe epilepsy with normal MRI; VBM, voxel-based morphometry; *T, T* score.

The VBM *z*-score maps demonstrated increased gray matter volume (GMV) only in the left amygdala of MTLE-NL group compared to controls (MNI coordinates: −24 −7 −21; *T* maximum = 3.69; number of contiguous voxels: 139). Details of GM volume increase are show in Figure 1.

### Amygdala volumetry

Individual volumetric analysis confirmed increased AVs in eight (12%) MTLE-NL patients.

From the five patients with unilateral AE, two had the increased amygdala ipsilateral to the epileptic focus, two contralateral, and one had bitemporal EEG focus. From the three patients with bilateral amygdala increase, two had the predominant side of increased volume ipsilateral and one contralateral to the interictal EEG abnormalities. Overall, from all patients with AE and defined epileptic focus, four (57%) had the increased or predominantly increased volume ipsilateral to the epileptic focus.

Five patients had borderline AE. None had significant decrease of amygdala volume.

### T2 relaxometry

T2 relaxometry demonstrated no amygdala hyperintense signal in any individual with significant AE.

### Clinical data

There were no clinical differences between the two groups (with and without AE) regarding sex, age, age of seizure onset, family history of epilepsy, history of initial precipitating injury, status epilepticus, duration of epilepsy, AED response or occurrence of GTCS in the previous year (Table 1). None of the patients with enlarged amygdala underwent surgery and therefore pathological specimens were not available.

**Table 1:** **Demographic and clinical data MTLE-NL patients with or without increased amygdala**.

	Normal amygdala (*n* = 48)	Increased amygdala (*n* = 8)	*p* Value
Sex	M = 26	M = 1	*p* = 0.081 (Pearson Chi-square)
Mean age (range)	41.7 (19–74 years)	47.6 (29–68 years)	*p* = 0.274 (*t*-test)
Mean age of seizure onset (range)	19.8 (2–48 years)	17.9 (8–47 years)	*p* = 0.702 (*t*-test)
Family history of epilepsy	29	3	*p* = 0.507 (Pearson Chi-square)
IPI (FS)	12 (3)	2 (0)	*p* = 0.78 (Pearson Chi-square)
SE	1	0	*p* = 0.708 (Pearson Chi-square)
Mean duration of epilepsy (range)	21.9 (1–50 years)	25.75 (15–48 years)	*p* = 0.144 (*t*-test)
Seizure remission	12 (25%)	1 (12.5%)	*p* = 0.585 (Pearson Chi-square)
Number of patients with SGTCS in the previous year	11 (23%)	1 (12.5%)	*p* = 0.657 (Pearson Chi-square)

*MTLE-NL, mesial temporal lobe epilepsy with normal MRI; M, male; IPI, initial precipitating injury; FS, febrile seizure; SE, status epilepticus; SGTCS, secondary generalized tonic-clonic seizures*.

### Cross-validation VBM group analysis

**Figure 2:**
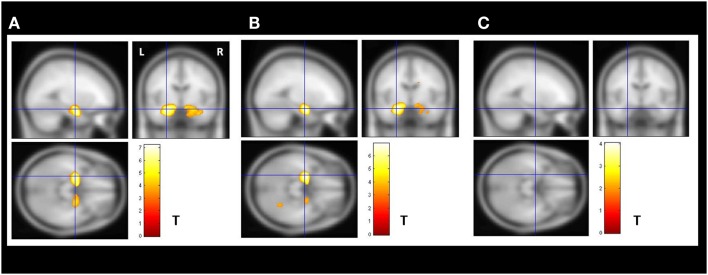
**Secondary VBM group analysis**. Secondary VBM analysis confirmed the finding of subgroups of MTLE-NL patients with or without amygdala enlargement detected by automatic volumetry. **(A)** VBM demonstrated bilateral increased amygdala gray matter volume in the enlarged amygdala group (*N* = 8); **(B)** VBM also detected increased amygdala gray matter volume in the subgroup of patients with borderline amygdala volumes (amygdala volumes *z*-score higher than 1.5 SD in the volumetric analysis; *N* = 14); **(C)** VBM analysis did not detected increase gray matter volumes in the subgroup of patients with normal amygdala defined by the volumetric analysis. VBM, Two-sample *t*-test, *p* < 0.05, FDR corrected, minimum cluster size of 30 voxels. MTLE-NL, mesial temporal lobe epilepsy with normal MRI; VBM, voxel-based morphometry; FDR, false discovery rate; *T, T* score.

The VBM *z*-score maps demonstrated bilateral increased amygdala GMV in the enlarged amygdala group compared to controls (Figure 2A).

The increased amygdala GMV was still present in the VBM *z*-score maps when comparing controls to the borderline AVs (Figure 2B).

The VBM analysis comparing controls to the patents with normal AVs did not show areas of increased gray matter (Figure 2C).

## Discussion

In this study we demonstrated an increase of the amygdala volume in 12% of patients with MTLE-NL using two different methods. The amygdala is known for its central role in emotional behavior and it plays an important function in epilepsy and epileptogenesis (13, 14). The involvement of the amygdala in MTLE has been largely investigated; however, its complete participation in MTLE is still unknown (14). Findings such as stimulation of the amygdala leading to experiential symptoms (15–17) as well as epileptiform discharges arising from the amygdala in intracranial EEG recordings (18) are corroborating evidence of the importance of this structure in MTLE. Studies with AVs in MRI-negative patients have already been done and incidental cases of unexpected AE have already been reported (14). Although the involvement of the amygdala in MRI-negative MTLE has already been suggested, this pattern was not observed in previous VBM studies, which could be explained by the heterogeneity among MRI-negative patients and small number of patients in previous studies.

The hypothesis that a subgroup of MTLE with amygdala involvement may exist was already raised before (14, 18). Recently, we demonstrated that a subgroup of patients with MTLE associated with HS and early epilepsy onset have increased AVs, and occasionally also hippocampal volumes, contralateral to the epileptogenic zone (12). However, the frequency of this finding and the clinical differences of patients with MTLE-NL with or without abnormal amygdala volume have not been evaluated. The results observed herein after individual automatic volumetry analysis and after secondary VBM analysis strengthen this hypothesis. Previous studies with MRI-negative epilepsies have shown that there is indeed heterogeneity of epileptic pathologies in these patients (3). Our finding probably points out the existence of a subgroup of MTLE-NL with amygdala abnormalities. However, the meaning of the increased volume of the amygdala remains unknown. We could hypothesize that it may represent dysplasia, dysgenesis, or other subtle structural abnormality. However only further studies with pathological correlation would help us understand this MRI finding.

Indeed, a series of 100 MTLE patients submitted to temporal lobectomy with amygdalectomy and minimal hippocampal resection showed similar outcomes as compared to a series of 100 MTLE patients submitted to temporal lobectomy with major hippocampectomy in the same institution (19, 20). This and other series, in addition to SEEG data (21, 22) supports the notion that some patients with MTLE may have a major amygdalar seizure focus and may not require removal of hippocampus. This would be particularly relevant for patients with normal MRI since these patients are at high risk for memory decline after removal of a normal appearing hippocampus on MRI. In a recent series of patients who were submitted to a tailored resection sparing the hippocampus they showed that 96.8% of patients did not have worsening of post-operative memory performance (23).

The main limitation of the present study is that we did not have ictal intracranial EEG recordings of these patients, nor surgical treatment with pathological specimens. However, this preliminary finding may give support for further investigations for defining MRI surrogate markers of amygdala pathology and thus, helping to identify patients who would benefit from a selective amygdala removal sparring the hippocampus and parahippocampus. In future studies, the amygdala volume should be investigated in the pre-operatory MRI of patients with MTLE-NL who will be submitted to anterior temporal lobe resections and special attention should be given to the pathology of amygdala in these individuals.

The results reported herein emphasize the possible role of the amygdala in MTLE-NL, suggesting that there might be, at least in some cases, a structural abnormality in the amygdala that may be involved in some patients with MRI-negative MTLE. The enlargement of the amygdala could be the source of the pathology of these individuals. Further studies are necessary but this preliminary finding could be of great importance not only in the understanding of MTLE-NL and also in the surgical treatment of this condition.

## Conflict of Interest Statement

Ana Carolina Coan, MD., has received support from FAPESP (Fundação de Amparo à Pesquisa do Estado de São Paulo). Marcia Elisabete Morita, MD, Ph.D., has received support from CAPES. Brunno Machado de Campos has received support from FAPESP. Clarissa Lin Yasuda, MD, Ph.D., has received support from FAPESP. Fernando Cendes, MD, Ph.D., has received support from FAPESP and Conselho Nacional de Desenvolvimento Científico e Tecnológico (CNPq).

## Footnotes

^1^http://surfer.nmr.mgh.harvard.edu/

^2^http://www.liv.ic.unicamp.br/~bergo/aftervoxel
